# Surface Induced Phenytoin Polymorph. 2. Structure Validation by Comparing Experimental and Density Functional Theory Raman Spectra

**DOI:** 10.1021/acs.cgd.9b00863

**Published:** 2019-09-18

**Authors:** Andrea Giunchi, Arianna Rivalta, Natalia Bedoya-Martínez, Benedikt Schrode, Doris E. Braun, Oliver Werzer, Elisabetta Venuti, Raffaele Guido Della Valle

**Affiliations:** † Department of Industrial Chemistry “Toso Montanari”, University of Bologna, Viale Risorgimento 4, I-40136 Bologna, Italy; ‡ Materials Center Leoben Forschung GmbH, Roseggerstraβe 12, 8700 Leoben, Austria; ⊥ Institute of Pharmaceutical Science, Department of Pharmaceutical Technology, University of Graz, 8010 Graz, Austria; § Institute of Solid State Physics, NAWI Graz, Graz University of Technology, Petersgasse 16, 8010 Graz, Austria; ∥ Institute of Pharmacy, University of Innsbruck, Innrain 52c, 6020 Innsbruck, Austria

## Abstract

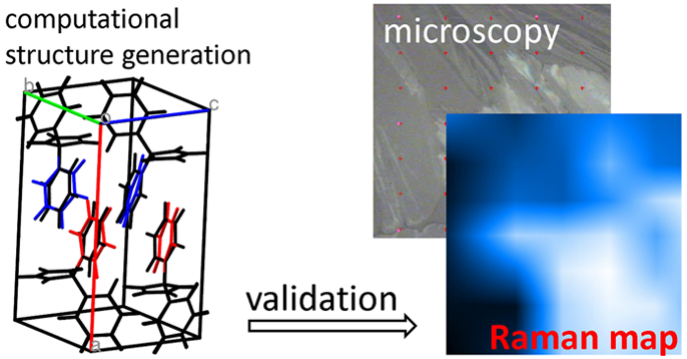

A method for structure solution in thin films that combines grazing incidence X-ray diffraction data analysis and crystal structure prediction was presented in a recent work (Braun et al. Cryst. Growth Des.2019, DOI: 10.1021/acs.cgd.9b00857). Applied to phenytoin form II, which is only detected in films, the approach gave a very reasonable, but not fully confirmed, candidate structure with *Z* = 4 and *Z*′ = 2. In the present work, we demonstrate how, by calculating and measuring the crystal Raman spectrum in the low wavenumber energy region with the aim of validating the candidate structure, this can be further refined. In fact, we find it to correspond to a saddle point of the energy landscape of the system, from which a minimum of lower symmetry may be reached. With the new structure, with *Z* = 4 and *Z*′ = 2, we finally obtain an excellent agreement between experimental and calculated Raman spectra.

## Abstract 2

Experimental low wavenumber Raman spectroscopy validates the presence of a non-centrosymmetric packing arrangement that is energetically very close to a centrosymmetric saddle point structure.

## 1 Introduction

Phenytoin is an anticonvulsant drug, also known by the commercial name of Dilantin. Only one crystal form of phenytoin has been fully characterized so far in the literature (form I). It has an orthorhombic lattice, space group *Pna*2_1_ (*C*_2*v*_^9^), with four molecules in the unit cell, all equivalent by symmetry (*Z* = 4, *Z*′ = 1).^2,3^ A second phase (form II) has been recently identified by grazing incidence X-ray diffraction (GIXD) on thin film samples deposited on silica substrates;^4^ it is therefore a surface induced polymorph (SIP), not yet fully characterized in the literature.

As discussed in the accompanying article,^1^ GIXD measurements on crystalline phenytoin films detected in all cases diffraction peaks characteristic of form I and, in some cases, additional peaks due to form II. By varying and optimizing the deposition conditions, it was possible to obtain enough peaks for a successful indexing, yielding the lattice parameters of form II, which was found to be monoclinic. Further analysis of the GIXD data, combined with crystal structure prediction (CSP) methods, gave a preliminary *P*2_1_/*c* (*C*_2*h*_^5^) structure with four molecules in the unit cell, all equivalent by symmetry (*Z* = 4, *Z*′ = 1).

The proposed *P*2_1_/*c* structure appeared plausible and reproduced the experimental diffraction pattern. Seeking further validation and full identification of such a structure, we decided to measure and calculate the Raman spectra of both phenytoin crystal forms in the low wavenumber region (up to ∼150 cm^–1^) which probes the lattice vibrations (phonon modes) that are extremely sensitive to the details of crystal packing. Indeed, the synergy between highly accurate density functional theory calculations with dispersion corrections (DFT-d) and low frequency Raman spectroscopy is very effective^5−7^ for polymorph identification. Analysis of the spectra, and comparison between experimental and calculated spectra, may thus be used to corroborate and validate any suggested structure.

Except for checking the chemical identity of the samples, we do not make use of high wavenumber frequencies characteristic of molecular bonds and functional groups since, being insensitive to the molecular packing, they are not relevant to our analysis.

In the present work, we have therefore used the microRaman technique to map areas of phenytoin films where distinct phases appear, to identify the lattice phonon pattern typical of each of them. Polarized Raman spectroscopy has been used to support the assignment of the observed features. The satisfactory match between experiments and calculations for phenytoin form I, both in unpolarized and polarized spectra, demonstrated the reliability of the computational approach. For form II, the same analysis has instead led us to revise the preliminary *P*2_1_/*c* structure, which is centrosymmetric. From a computational point of view, the structure is found to correspond to an energy saddle rather than a minimum, and moreover the calculated spectra display a very poor agreement with the experiments. By perturbing the saddle configuration, a new non-centrosymmetric *Pc* minimum is reached, with two nonequivalent pairs of molecules in the unit cell (*Z* = 4, *Z*′ = 2). Such a minimum, although extremely close to the *P*2_1_/*c* structure, is characterized by a different set of Raman symmetry selection rules and thus yields very different spectra, which finally do account for the experimental results. Accordingly, the computed GIXD pattern is also very satisfactory and actually represents an improvement to that of the preliminary *P*2_1_/*c* structure.

## 2 Experimental and Theoretical Methods

### 2.1 Raman Spectroscopy

Various samples were investigated by Raman spectroscopy for the purposes of this work: (1) commercial phenytoin from Sigma-Aldrich, available as a crystalline powder certainly attributable to form I;^2,3^ (2) elongated needle-like single crystals obtained by slow evaporation from solutions in ethanol (EtOH); (3) films obtained by drop casting a THF solution on heated silica glass substrates,^4^ following the procedure described in the accompanying article.^1^

All samples were characterized by Raman spectroscopy, limiting the analysis to the low wavenumber energy range (up to ∼150 cm^–1^). A flexible molecule such as phenytoin is expected to display over this range also intramolecular vibrations, like for instance the hindered rotations of the phenyl rings, which mix with the lattice phonons. The spectral pattern in this region is unique for each crystal structure.

Raman spectra were recorded with a Horiba Jobin Yvon T64000 spectrometer. The spectrometer is equipped with three monochromators in double subtractive configuration to achieve detection down to ∼10 cm^–1^ and is coupled to an Olympus BX40 confocal microscope equipped with 100×, 50×, 20×, and 10× objectives. This allows collecting information on the polymorphic behavior in crystal domains of micrometric dimensions both in bulk and film systems. Confocality allowed us to achieve a lateral resolution below 1 μm with the 100× objective and a nominal field depth ranging from about 7 to 900 μm. The excitation wavelength was from a Kr^+^ laser tuned at 647.1 nm. The incoming power was reduced with a neutral filter whose optical density was selected in each experiment to prevent crystal damage, the actual power focused on the sample being anyway less than 1 mW (estimated power density 2500 W/cm^2^).

A half-wave plate was used to rotate the polarization of the incident light, while a wire grid polarizer selected the polarization of the scattering. X-ray indexing on crystal faces, morphology, and extinction directions allowed us to orient the crystal specimens for measurements in polarized light. For unpolarized measurements on crystals (not oriented) and powder, no polarization scrambler was applied to the laser beam.

### 2.2 Computational Methods

Phenytoin polymorphs were theoretically described by density functional theory (DFT) calculation with the VASP software (Vienna Ab initio Simulation Package), and with the Perdew–Burke–Ernzerhof (PBE) exchange correlation functional in combination with the projector-augmented wave (PAW) pseudopotentials.^8−13^ The effects of van der Waals (vdW) interactions were included with the computationally efficient pairwise D3-BJ method by Grimme.^14^ Energy convergence could be achieved for the two polymorphs by using a plane wave energy cutoff of 800 eV. Monkhorst–Pack *k*-point samplings of 2 × 1 × 1 and 1 × 1 × 2 for forms I and II were used, respectively.

The *k*-point grid was selected in such a way as to obtain the same density of sampling points in each direction and is thus dependent on the lattice parameters. Raising the cutoff energy from 800 to 1200 eV caused energy changes below 1 meV per atom, while the differences in energies between the adopted *k*-point grid and denser ones were below 1 meV per atom. Initial experimental atomic coordinates were fully relaxed toward the nearest stationary point using the GADGET package,^15^ halting the optimization when residual forces fell below 1 meV/Å.

Lattice parameters were constrained to their experimental values since we have found that unconstrained computed unit cell volumes are smaller than experimental volumes.^1^ This was expected because DFT calculations are effectively at 0 K and thus neglect the thermal expansion, which due to vibrational anharmonicity tends to lower the frequencies.^16^ By computing the frequencies at the stationary point with the experimental unit cell parameters, we account for these effects.

Vibrational modes for *k* = 0 were determined with the phonopy package^17^ in combination with VASP, by computing and then diagonalizing the “dynamical matrix” given by the second derivatives of the crystal energy with respect to the atomic coordinates. The eigenvectors yield the vibrational displacements of the atoms, also involved in the calculations of Raman intensities. By analyzing the transformation properties of the eigenvectors, we deduce the symmetry species of the modes, while the square roots of the eigenvalues correspond to the frequencies, also used to check the stability of the stationary point.

As a necessary mathematical condition for *local* stability of the lattice with respect to displacements of the atoms, the potential energy surface must in fact be convex around the stationary point reached by the optimization, or, equivalently, all vibrational frequencies must be real and positive (*positive definite* dynamical matrix^16^). Imaginary frequencies indicate that the stationary point is a saddle instead of a minimum. Starting from such a saddle, it may be possible to reach nearby minima by perturbing the system along the mode (or modes) with imaginary frequency.

Polarizability tensors α_*ij*_ for each crystal mode were obtained by using the Python program vasp_raman.py,^18^ which uses the VASP code as backend. Polarized Raman spectra on oriented crystals probe single α_*ij*_ components, while appropriate averages have to be computed when a distribution of different orientations is present. Unpolarized spectra on powder samples, for example, involve an average over all possible orientations in three dimensions and thus follow the same rules as gases.^19^

## 3 Results and Discussion

### 3.1 Experimental Results (Raman Spectra)

#### 3.1.1 Form I

**Figure 1:**
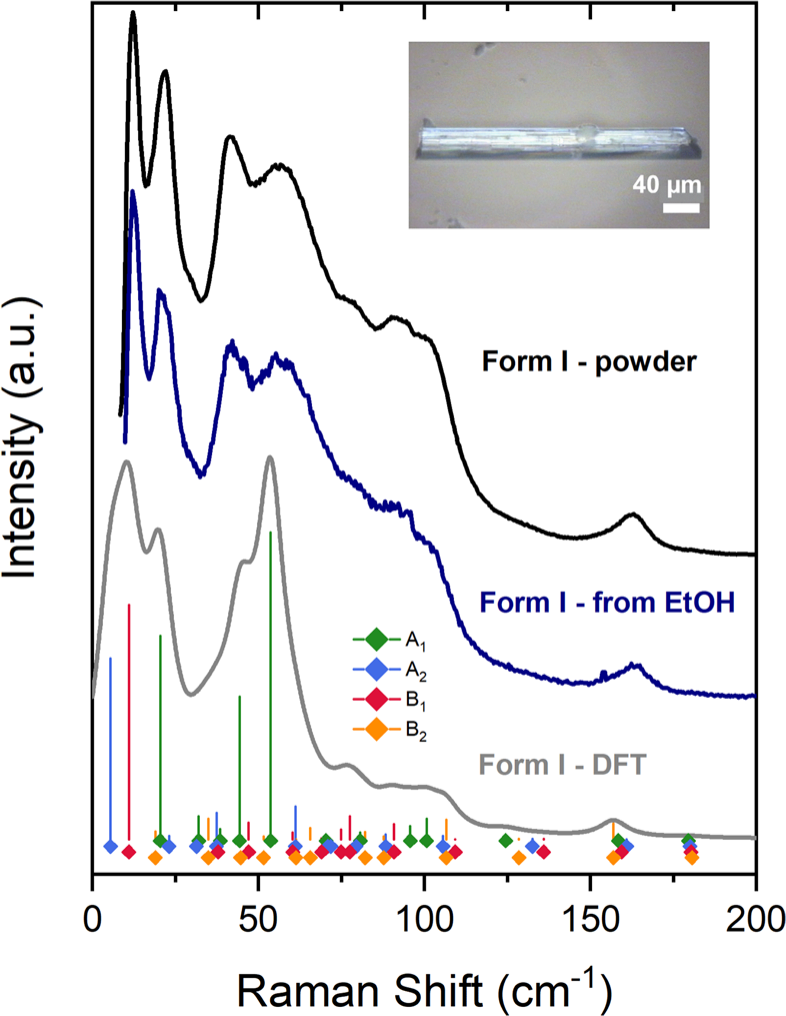
Upper traces: Unpolarized Raman spectrum of the commercial powder (form I) and of a single needle-like crystal obtained by deposition from EtOH (sample image shown in the inset) in the wavenumber range 15–200 cm^–1^. Lower trace: computed unpolarized spectra for form I. Position and height of the vertical bars represent computed frequencies and intensities of the vibrational modes. Mode symmetries are labeled by colors, as indicated in the figure.

Unpolarized Raman spectra in the wavenumber interval 15–200 cm^–1^ of the commercial powder and of a needle-like single crystal are shown in Figure 1, along with the computed frequencies and intensities which will be discussed in the following. The two spectra clearly correspond to the same crystal structure, and since the powder certainly belongs to the more common form I, we deduce that the needle belongs to the same form. X-ray indexing of phenytoin needles^20^ obtained from EtOH and displaying the same morphology have shown that the needle axis lies in the direction of the crystal axis *a*. This observation is in agreement with the literature finding for orthorhombic molecular crystals,^21^ for which the long side of the crystal, that is, the direction of fastest growth, is usually parallel to the shortest axis (i.e., *a*).

**Figure 2:**
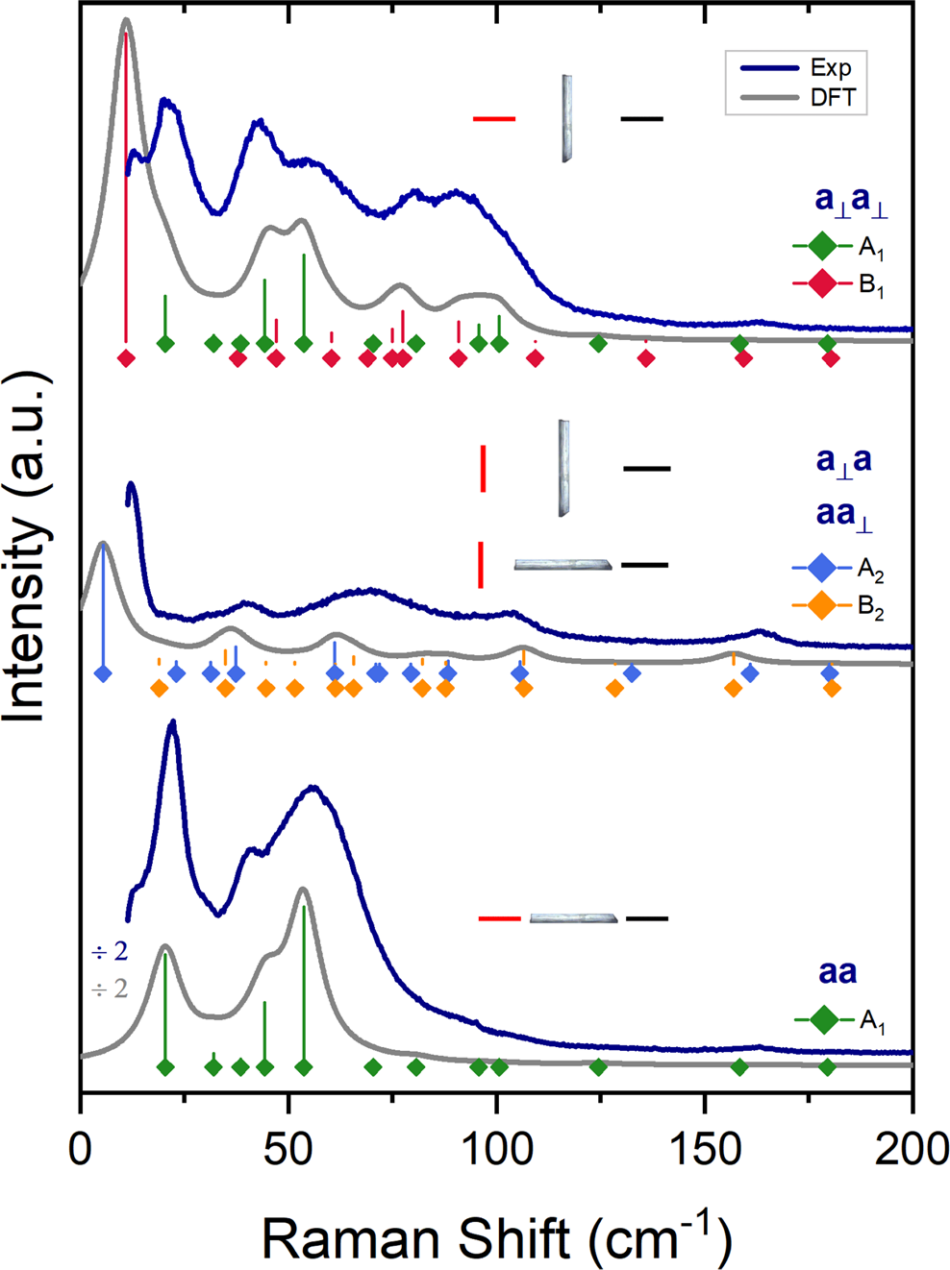
Colored traces: Polarized Raman spectra for a single needle-like crystal of form I in the wavenumber range 15–200 cm^–1^. Horizontal or vertical bars in the insets indicate the direction of the polarization vector of incident and scattered light with respect to the needle axis. The notation *a* or *a*_⊥_ indicates a direction parallel or perpendicular to the needle, which grows along the crystal axis *a*. Gray traces: Calculated spectra evaluated as discussed in the text, for clarity all shown with a downward intensity shift. Position and height of the vertical bars represent computed frequencies and intensities of the vibrational modes. Mode symmetries are labeled by colors, as indicated in the figure.

Polarized Raman spectra of the same crystal recorded in a backscattering geometry are shown in Figure 2. The sample is placed on the stage with the needle axis, which corresponds to one of the extinction directions and identifies with the crystal axis *a*, either parallel or perpendicular to the polarization direction of the analyzer (output polarizer), which, as indicated in the figure, had a fixed orientation, while the polarization direction of the impinging radiation could be rotated. Accordingly, the polarization of exciting and scattered light beams is indicated by the labels *a* or *a*_⊥_, depending on their orientation either parallel or perpendicular to the crystal axis *a*. As expected from theory, spectra recorded in cross-polarization, i.e., *aa*_⊥_ or *a*_⊥_*a* spectra, were found to look identical, and therefore only one of them is shown in Figure 2.

For the purposes of labeling the vibrational symmetry species^22^ in the *C*_2*v*_^9^ factor group, it is necessary to specify that the *C*_2_ axis lies along *b* and that we have arbitrarily chosen the σ_v_ and σ_v_^′^ planes to be on *bc* and *ab*, respectively (the opposite choice would of course be possible). This information on the symmetry of the crystal is sufficient to interpret and assign the most important spectral features. As mentioned above, polarizations parallel to the needle are aligned to the orthorhombic axis *a*, while those perpendicular to it (*a*_⊥_) are along some unknown combination of *b* and *c*.

Therefore, *aa* spectra probe modes of *A*_1_ symmetry and derive their intensities from the α_*aa*_ component of the polarizability tensor. The bands observed in *a*_⊥_*a*_⊥_ spectra arise from the α_*bb*_, α_*cc*_, and α_*bc*_ polarizability components, and correspond to modes of symmetry *A*_1_ and *B*_1_. Finally, *aa*_⊥_ (or *a*_⊥_*a*) spectra involve α_*ab*_ and α_*ac*_ polarizability components, thus probing modes of symmetries *B*_2_ and *A*_2_, respectively.

#### 3.1.2 Form II

**Figure 3:**
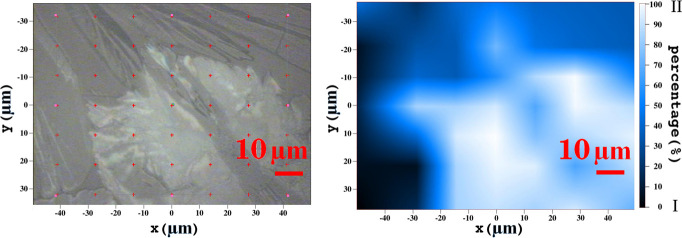
Optical image (left) and Raman map (right) of a selected film sample. The Raman map refers to the *XY* grid of points drawn over the optical image, with a color scaling from extremely dark (form I) to extremely light blue (form II).

**Figure 4:**
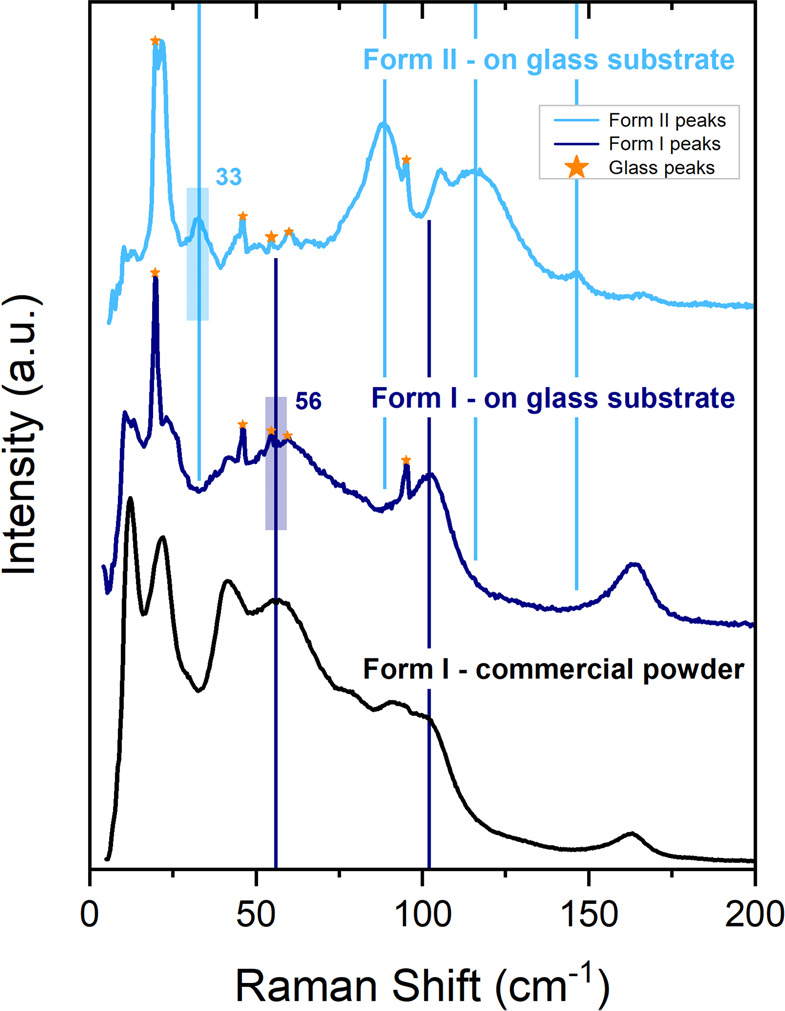
Low wavenumber Raman spectra of phenytoin commercial powder, and at two different points of a selected film prepared by drop casting. Peaks indicated with stars are due to the glass substrate. Shaded areas indicate spectral windows chosen to quantify the proportion of the two phases.

Films prepared by drop casting adopt very nonuniform morphologies, as shown by the optical image of a typical sample in Figure 3. For this sample, we have used the confocal microscope to acquire Raman spectra at all points of the grid superimposed over the image and found different spectral patterns, as illustrated in Figure 4, where the spectrum of the commercial powder, which belongs to form I, is also shown. Spectra with lattice peaks precisely matching those of the commercial powder are found at some grid points, where only form I is therefore present. At other grid points, we record spectra where none of the characteristic peaks of form I are visible. It is therefore possible to infer that only the different form II (pure SIP) is present at these areas. At yet other points, we find combinations of the two detected spectra, which clearly correspond to mixtures of the two forms (phase coexistence or mixing). Analogous behavior is found for other samples.

A full map of the relative proportions of the two forms at the various grid points has thus been deduced from the relative intensity of appropriate spectral windows typical of the two forms. We have used the window 53–59 cm^–1^ for form I and 30–36 cm^–1^ for form II, as indicated by the shaded areas in Figure 4, resulting the concentration map shown in Figure 3 (right panel). As expected, we observe a close correspondence between the Raman map and the optical image (i.e., between spectrum and morphology), with form II corresponding to whitish powder-like portions of the sample, and form I to darker fibrous portions.

To check for the chemical identity of the sample, we have recorded the Raman spectra on the intramolecular vibration region (500–1250 cm^–1^), reported in Figure S1 of the Supporting Information, where the few spectral differences detected between the two forms in this range are indicated.

### 3.2 Computational Results

#### 3.2.1 Form I

Starting from the experimental structure,^3^ we relaxed it to the equilibrium structure with VASP and then computed vibrational frequencies, eigenvectors, and Raman intensities. All frequencies were computed to be positive, proving that the found structure is indeed a stable minimum. Experimental and calculated unpolarized Raman spectra of form I are shown in Figure 1. The calculated spectra were obtained at 293 K (i.e., using the experimental lattice parameter corresponding to this temperature), for an exciting laser line at 647.1 nm. They are the sum of Lorentzian bands with the computed frequencies and intensities, and full widths at half-maximum (fwhm) chosen to match the experimental widths and fixed at 5 cm^–1^. The agreement between calculated and experimental spectra is fair, with the exception of the lowest peak around 10 cm^–1^, for which the calculated intensity exceeds the measured peak height. We consider such a discrepancy as an artifact due to the subtractive monochromator configuration of the experimental setting, which is designed to cut intensities in the vicinity of the laser exciting line.

Experimental and calculated polarized Raman spectra for form I are shown in Figure 2. As already mentioned, about the *a*_⊥_ orientation we only know that it must lie in the *bc* plane, not necessarily corresponding to a crystal axis. In the calculation of the spectra, we have therefore averaged (i.e., marginalized) the intensity contributions over the unknown rotation around the *a* axis, obtaining for the different polarized spectra the intensities *I*_*aa*_ ∝ α_*aa*_^2^, *I*_*aa*_⊥__ ∝(α_*ab*_^2^ + α_*ac*_^2^)/2 and *I*_*a*_⊥_*a*_⊥__ ∝ (3α_*bb*_^2^ + 4α_*bc*_^2^ + 2α_*bb*_α_*cc*_ + 3α_*cc*_^2^)/8, where each term involves the expected α_*ij*_ polarizability components. Except for the *aa* spectrum, which is angle independent, the intensities for a specific but unknown rotation would be constructed with angle-dependent combinations of the same components. Details on the derivation of the previous formulas and calculated intensities are reported in Section 2.1 and Table S1 of the Supporting Information, respectively. An almost perfect agreement between calculations and experiments is found for the *aa* spectrum. This spectroscopic result confirms the deduction that the long side of the crystal is parallel to *a* axis. The good match between calculated and experimental spectra, both unpolarized and polarized, furthermore validates the computational method. The agreement for the polarized spectra, in particular, is a novel, very stringent test since it indicates that the vibrational eigenvectors are well described.

#### 3.2.2 Form II

**Figure 5:**
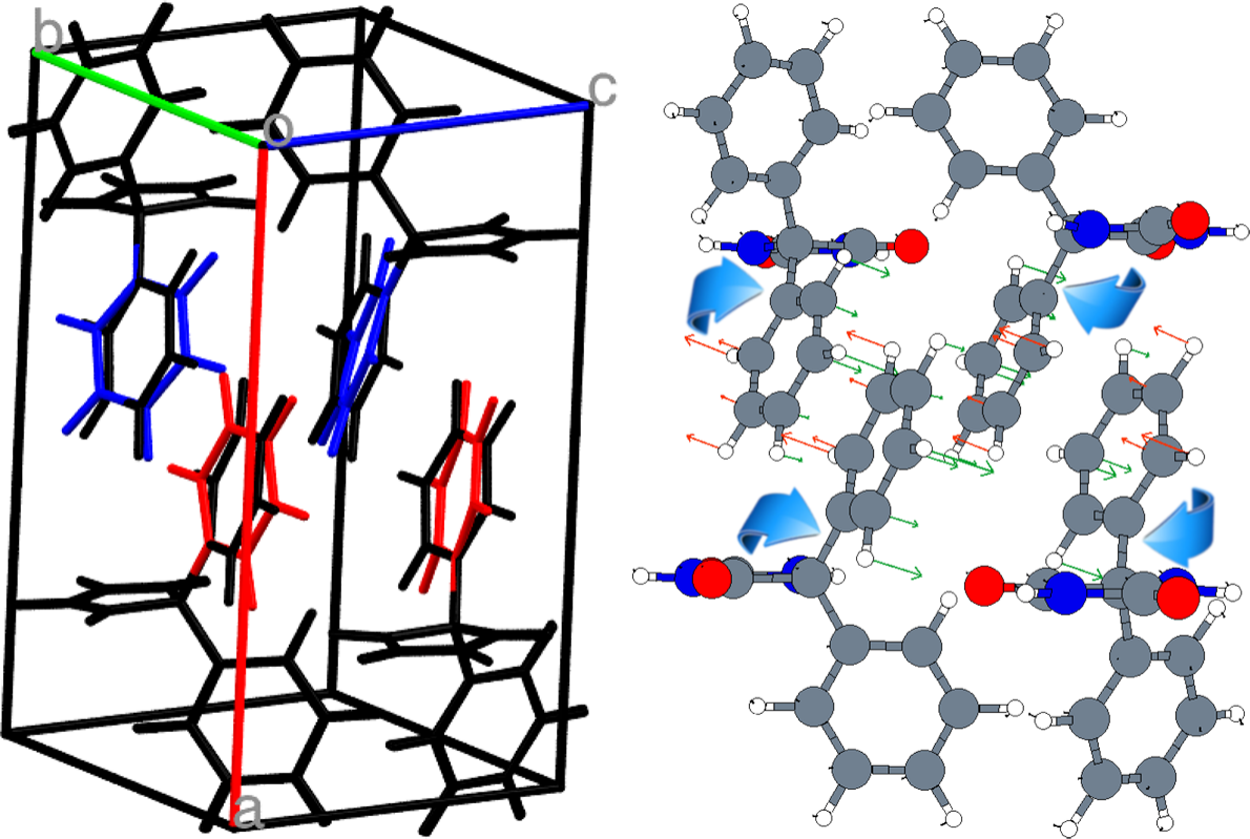
Left panel: Overlapped structures of the two DFT-d stationary points of form II. *P*2_1_/*c* (black) and *Pc* (colored) structures are respectively an energy saddle and a minimum and differ for the rotation of the phenyl groups. Molecules with the same color are equivalent by symmetry. Right panel: Atomic displacements (eigenvectors) of the *B*_u_ mode with imaginary frequency.

**Figure 6:**
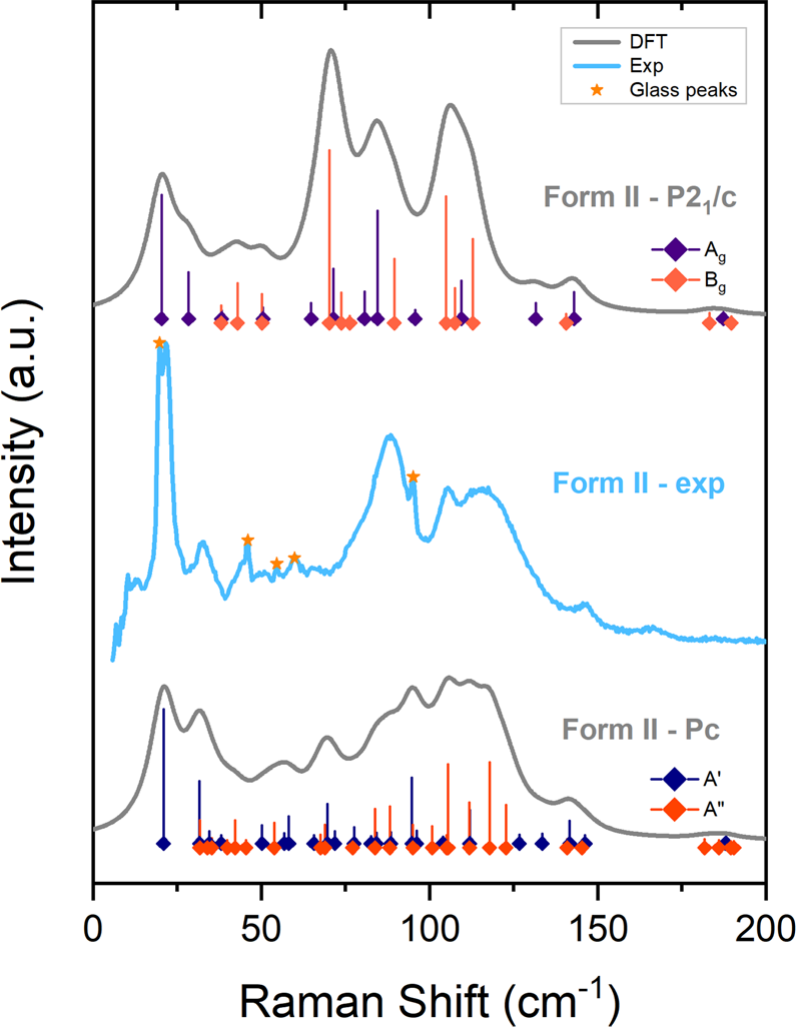
Experimental unpolarized Raman spectra for form II of phenytoin (colored trace) and computed powder spectra for the *P*2_1_/*c* and *Pc* computed structures (gray traces). Position and height of the vertical bars represent computed frequencies and intensities of the vibrational modes. Mode symmetries are labeled by colors, as indicated in the figure. As discussed in the text, the *A*_g_ and *B*_g_ modes of *P*2_1_/*c* are two strict subsets of *A*′ and *A*″ of *Pc*, respectively.

As already discussed, previous GIXD measurements and CSP calculations gave a *P*2_1_/*c* (*C*_2*h*_^5^) crystal structure that reproduced quite well the experimental diffractogram.^1^ By relaxing this structure to the nearest stationary point with VASP, and then computing the vibrational modes, however we have now discovered that the experimental spectrum was not properly reproduced, as it can be seen when comparing the experimental and computed unpolarized Raman spectra shown in Figure 6. More importantly, we have found an imaginary frequency for an intramolecular mode of *B*_u_ symmetry, i.e., with atomic displacements that are antisymmetric with respect to both the inversion and *C*_2_ operations, indicating that the preliminary *P*2_1_/*c* packing corresponds to a saddle point rather than to a genuine energy minimum. A graphical representation of this mode is shown in Figure 5.

By perturbing the system along the eigenvector of the imaginary frequency, all symmetry operations of *P*2_1_/*c* group except the glide plane are lost, and a new *Pc* (*C*_*s*_^2^) minimum energy structure is obtained (besides a shift of the origin, the result is same regardless of the + or – sign of the perturbation).

The *Pc* minimum is only slightly more stable than the original *P*2_1_/*c* saddle point (Δ*E* = 0.07 kcal per mole of phenytoin) and is geometrically extremely close to it. So close, in fact, that the standard crystallographic tool PLATON^23^ used in the CSP search for phenytoin^1^ to check for higher symmetries, with the default distance tolerance for the identification of atoms equivalent by symmetry (0.25 Å), converts the *Pc* structure back to the *P*2_1_/*c* one. This symmetry check therefore had to be disabled for the *Pc* structure, which could be located only by detecting the lack of local stability of the system diagnosed through the occurrence of the imaginary frequency, accompanied by eigenvector following. The missing of a slightly different structure of lower symmetry could be a problem, which might remain undetected, in CSP searches.

Despite their close structural and dynamical similarities, the two structures can be promptly distinguished on the basis of their vibrational properties, because in the *Pc* packing all the modes become Raman active, as a result of the symmetry lowering with loss of inversion. For this reason, the calculated *Pc* spectrum displays more bands and, as shown in Figure 6, agrees much better with the experiment.

By analyzing the structures in detail, we find that the transformation leading from the *P*2_1_/*c* (*C*_2*h*_^5^) to the *Pc* (*C*_*s*_^2^) structure involves a slight rotations of the phenyl groups in two opposite directions (see Figure 5). As a consequence, the inversion and *C*_2_ screw axis symmetries of *C*_2*h*_^5^ are lost, while the glide plane symmetry is preserved. Depending on their parity with respect to the latter symmetry operation, the four irreducible representations of the *C*_2*h*_^5^ group merge into the two *C*_*s*_^2^ representations, with the correlation scheme *A*_g_ + *B*_u_ → *A*′, *A*_u_ + *B*_g_ → *A*″. By examining the calculated intensities in Figure 6, in fact, we find that *A*_g_ or *B*_g_ bands computed for the *P*2_1_/*c* structure usually have corresponding *A*′ or *A*″ bands for the *Pc* structure which, however, display many additional bands, which are those arising from the *A*_u_ or *B*_u_ Raman inactive *P*2_1_/*c* modes. The intensities calculated for the *Pc* and *P*2_1_/*c* structures of form II are listed in Tables S2 of the Supporting Information.

## 4 Summary and Conclusions

An accompanying article of the present collaboration group^1^ reported on the solution of the crystallographic structure of the surface induced phase (SIP) of phenytoin (thin films known as phase II). This was determined by combining grazing incidence X-ray diffraction (GIXD) experiments with crystal structure prediction (CSP) calculations. Peak positions in a diffractogram depend solely on the unit cell parameters, while peak intensities depend on the positions of the atoms within the cell. For form II, the lattice parameters could be determined, whereas the atomic coordinates could not be obtained because of the very limited number of peaks in the thin film diffractogram. This, however, was sufficient to exclude (or possibly confirm) any proposed crystal structure. A large number of hypothetical structures was therefore generated and then optimized with CSP calculations using suitable molecule–molecule interaction models. Nearly 100 of the most stable structures were then used as starting points for DFT calculations with dispersion corrections (DFT-d). Among the optimized DFT-d stationary point configurations, possible candidate structures for forms I (bulk) and II (SIP) were finally identified by matching computed and experimental lattice parameters and further screened by comparing computed and experimental GIXD patterns. The best candidate for form I corresponded to the known crystallographic structure of bulk phenytoin, while the best candidate for form II reasonably matched the experimental lattice parameters and GIXD pattern and was therefore considered as a preliminary structure.

In the present work, aiming to validate the candidate structure for form II,^1^ we have investigated the experimental and calculated Raman spectra of the two crystal forms in the low wavenumber region which probes the lattice vibrations. These, being extremely sensitive to the details of crystal packing, represent the fingerprint of the structure. The comparison between experimental and calculated spectra may thus be used to validate any proposed structure. Vibrational properties are obtained by evaluating and then diagonalizing the dynamical matrix of the system. The computed frequencies can be compared to their experimental equivalents and also can be used to check the stability of the stationary point. Imaginary frequencies in fact indicate that the lattice is at a saddle point, from which one may reach nearby minima by following the modes with imaginary frequencies.

For form I, the experimental structure was correctly reproduced by the best CSP structure, which was confirmed to correspond to a stable minimum and gave computed Raman spectra in excellent agreement with the experiments. These results for the known structure successfully validate the computational method.

For form II, the best CSP structure, although able to reproduce the GIXD measurements, instead gave computed Raman spectra which did not match the experiments and furthermore corresponded to a centrosymmetric saddle point structure with an imaginary frequency. Descent from the saddle reached a non-centrosymmetric minimum, also with the correct GIXD pattern. The saddle and the minimum, although very close, are however characterized by different Raman symmetry selection rules. For the centrosymmetric saddle, the rule of mutual exclusion in fact holds, and no vibrational mode can be both infrared and Raman active, whereas all modes can be Raman active for the non-centrosymmetric minimum. The Raman spectra computed for the minimum, notwithstanding its structural similarity to the saddle point, are therefore very different and actually in excellent agreement with the experimental pattern, thus confirming that the structure is correct.

Convergence to saddle structures of too high symmetry could occur in many CSP searches. Detection of these saddles is often not attempted at DFT-d for hundreds of structures, although with increasing computer power it could be systematically performed by computing the vibrational spectrum and checking for imaginary frequencies. A related problem, in the opposite direction, was often noticed in the last decades of the 20th century, when it became clear that many of the published X-ray structural studies gave erroneous structures with too low crystallographic symmetry.^24^ This problem was eventually solved when software tools like PLATON^23^ were developed to routinely check the crystallographic coordinates for missing symmetries.^25^ By far the most common case is precisely the missing of an inversion center, which is also the most serious case because it leads to incorrect crystal property predictions. Reliable detection of the inversion in borderline situations presents problems even now,^24^ since PLATON (used by us to discover the space group of the predicted structures) needs to allow for noise in the atomic coordinates. Fortunately, as we have demonstrated in practice, an analysis of the Raman and/or infrared spectra may easily reveal the presence (or absence) of the inversion and thus conclusively validate a structure.

## Supporting Information Available

The experimental section includes the Intramolecular Raman spectra of phenytoin form I and form II. The computational section includes the Raman intensities of the bulk and the thin film form (PDF)
