# Supplementary material for: Surface Induced Phenytoin Polymorph. 2. Structure Validation by Comparing Experimental and Density Functional Theory Raman Spectra
Source: Cryst Growth Des. 2019 Sep 18;19(11):6067–73. doi: 10.1021/acs.cgd.9b00863 (PMC8016182; doi:10.1021/acs.cgd.9b00863)
Supplement: Supplementary file 1 — cg9b00863_si_001.pdf [file cg9b00863_si_001.pdf]

Supporting Information

Surface induced phenytoin polymorph part II: Structure validation by comparing experimental and DFT Raman spectra

Andrea Giunchi, Arianna Rivalta, Natalia Bedoya-Martínez, Benedikt Schrode, Doris E. Braun,*
Oliver Werzer,* Elisabetta Venuti, and Raffaele Guido Della Valle*

E-mail: doris.braun@uibk.ac.at; oliver.werzer@uni-graz.at; raffaele.dellavalle@unibo.it

Contents

1 Experimental **2**

1.1 Intramolecular Raman spectra of phenytoin form I and II 2

2 Computational **3**

2.1 Raman intensities for the orthorombic form I 3

2.2 Raman intensities for form II calculated with structures Pc and $P2_1/c$. . . . 4

1 Experimental

1.1 Intramolecular Raman spectra of phenytoin form I and II

The intramolecular Raman spectra of phenytoin form I and form II appear very similar and this confirms that the molecules assume virtually the same conformation in both polymorphs. Slightly different spectral features are observed in the energy range 950–1050 cm^{-1}, corresponding to CC and CH bending vibrations, which could be ascribed to the different H-bonding patterns of the two structures. Also, the so-called fingerprint range below 700 cm^{-1}, were mostly torsional modes are detected, displays some band shifts.

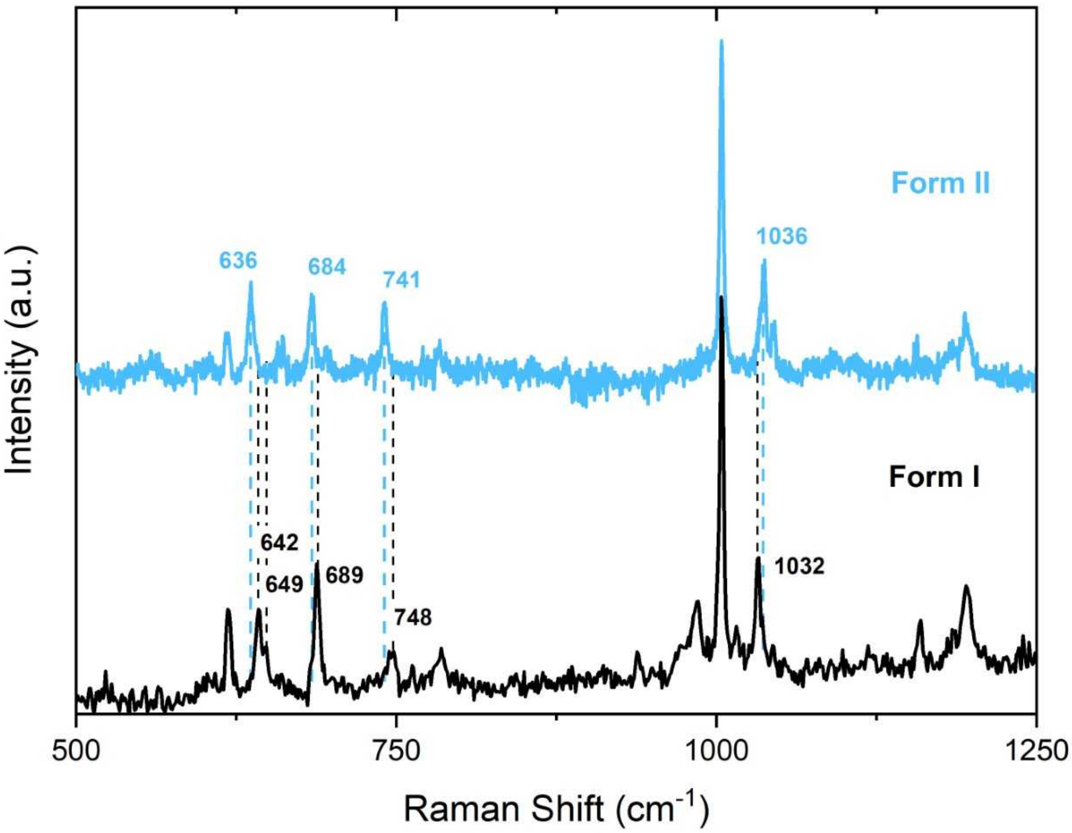

Figure S1: Raman spectrum of phenutoin forms I and II in the wavenumber interval 500-1250 cm^{-1}, where the intramolecular vibrations are detected. The vertical bars identify the bands which fall at different wavenumbers in the two polymorphs.

2 Computational

2.1 Raman intensities for the orthorombic form I

Polarized crystal Raman spectra (Figure 2 of main text and Table S1) with input and output polarizers aligned on the axes i and j have intensities $I_{ij} \propto \alpha_{ij}^2$ controlled by a single ij component of the polarizability tensor $\boldsymbol{\alpha}$. As discussed in the main text of the paper, for form I of phenytoin (orthorhombic) we have recorded polarized Raman spectra but we had only partial information about the identity of the crystallographic axes a, b, c in the needle-like sample. The shortest crystal axis a was certainly along the needle axis, whereas nothing was known about the axes b and c (except that they are perpendicular to a and to each other). In the measurements the two polarizers were chosen to be oriented either either parallel or perpendicular to the needle axis a and thus have direction either $\boldsymbol{a}$ or $\boldsymbol{a}_\perp = \boldsymbol{b}\sin\phi + \boldsymbol{c}\cos\phi$, were $\boldsymbol{a}$, $\boldsymbol{b}$, $\boldsymbol{c}$ indicate the direction of the three axes (three orthonormal vectors) and ϕ is the (unknown) angle between $\boldsymbol{a}_\perp$ and $\boldsymbol{b}$.

The correlate the measured intensities (I_{aa}, $I_{aa_\perp}$, $I_{a_\perp a_\perp}$) to the computed α_{ij} components of the polarizability tensor (α_{aa}, $\alpha_{ab} = \alpha_{ab}$, $\cdots$) we need to transform the polarizability derivatives $\boldsymbol{\alpha}$ from the crystal reference frame ($\boldsymbol{a}$, $\boldsymbol{b}$, $\boldsymbol{c}$) to the laboratory frame ($\boldsymbol{a}$, $\boldsymbol{a}_\perp$, $\boldsymbol{a} \times \boldsymbol{a}_\perp$). We must thus evaluate $\boldsymbol{R}\boldsymbol{\alpha}\boldsymbol{R}^\dagger$ were $\boldsymbol{R}$ is the rotation matrix between the crystal and laboratory frames which, for this specific case, is just a simple rotation by an angle ϕ around the $\boldsymbol{a}$ axis. The resulting intensities are

$$I_{aa} = \alpha_{aa}^2$$

$$I_{aa_\perp} = (\alpha_{ab}\sin\phi - \alpha_{ac}\cos\phi)^2$$

$$I_{a_\perp a_\perp} = (\alpha_{bb}\sin^2\phi - 2\alpha_{bc}\sin\phi\cos\phi + \alpha_{cc}\cos^2\phi)^2$$

The Raman spectra depend on the unknown angle ϕ and in theory we could try to deduce ϕ by fitting calculated and experimental spectra. However, this procedure would be very uncertain, and we prefer to average over all possible values of ϕ (from 0 to 2π), obtaining the estimated intensities reported in the main text

$$I_{aa} = \alpha_{aa}^2$$
$$I_{aa_\perp} = \frac{\alpha_{ab}^2 + \alpha_{ac}^2}{2}$$
$$I_{a_\perp a_\perp} = \frac{3\alpha_{bb}^2 + 2\alpha_{bb}\alpha_{cc} + 4\alpha_{bc}^2 + 3\alpha_{cc}^2}{8}$$

The unpolarized intensities (Figure 1 of main text and Table S1) are finally obtained by removing the polarizers, *i.e.* by summing on a and $a_\perp$: $I_{\text{unpol}} = I_{aa} + I_{a_\perp a} + I_{aa_\perp} + I_{a_\perp a_\perp}$, where $I_{a_\perp a} = I_{aa_\perp}$.

2.2 Raman intensities for form II calculated with structures Pc and $P2_1/c$.

As mentioned in the main text, unpolarized spectra on powder sample (Table S2 and Figure 6 of main text) involve an average over all possible rotations around the three Cartesian axes 1, 2, 3 and thus follows the same rules as gases, yielding

$$I_{\text{powder}} = \frac{45\alpha^2 + 7\beta^2}{45}$$

where α and β are the spherical part and the anisotropy of the polarizability

$$\alpha = \frac{a_{11} + a_{22} + a_{33}}{3}$$
$$\beta^2 = \frac{6\left(a_{12}^2 + a_{13}^2 + a_{23}^2\right) + \left(a_{11} - a_{22}\right)^2 + \left(a_{11} - a_{33}\right)^2 + \left(a_{22} - a_{33}\right)^2}{2}$$

Table S1: Calculated wavenumbers of the lowest energy modes of phenytoin form I. Symbols indicate symmetry and correspond to those used in Figures 1 and 2 of main text. For each mode we also report translational, rotational and internal components (%T, %R and %I), unpolarized (Figure 1) and polarized (Figure 2) Raman intensities for a needle-like sample, infrared and Raman intensities for a powder (given for the sake of completeness). Intensities of inactive modes for the given polarization and symmetry are indicated by hyphens.

Sym	Freq	%T	%R	%I	Needle Raman intensity				Powder	
	(cm^{-1})				I_{unpol}	I_{aa}	$I_{aa_\perp}$	$I_{a_\perp a_\perp}$	I_{IR}	I_{Raman}
A_1	20.4	16	74	10	0.663	0.549	–	0.113	0.001	0.256
	32.0	22	20	58	0.075	0.059	–	0.016	0.002	0.031
	38.5	56	26	18	0.034	0.030	–	0.004	0.024	0.013
	44.3	3	71	26	0.464	0.312	–	0.153	0.017	0.226
	53.7	1	25	74	1.000	0.785	–	0.215	0.013	0.404
	70.4	7	51	42	0.008	0.006	–	0.002	0.121	0.004
	80.7	3	14	83	0.023	0.017	–	0.006	0.032	0.010
	95.7	66	5	29	0.044	0.002	–	0.042	0.002	0.055
	100.6	23	0	77	0.067	0.003	–	0.063	0.006	0.082
	124.5	0	6	94	0.013	0.006	–	0.006	0.002	0.009
	158.4	0	2	98	0.002	0.001	–	0.002	0.132	0.002
	179.5	0	0	100	0.002	0.000	–	0.002	1.000	0.001
B_1	11.0	31	28	41	0.763	–	–	0.763	0.003	1.000
	37.9	3	79	18	0.001	–	–	0.001	0.040	0.001
	47.0	43	44	13	0.054	–	–	0.054	0.004	0.071
	60.3	56	8	36	0.023	–	–	0.023	0.009	0.030
	69.0	24	24	52	0.004	–	–	0.004	0.020	0.005
	74.9	9	3	88	0.032	–	–	0.032	0.012	0.041
	77.5	8	33	59	0.075	–	–	0.075	0.002	0.098
	90.8	22	47	31	0.049	–	–	0.049	0.011	0.064
	109.3	1	25	74	0.001	–	–	0.001	0.001	0.001
	135.9	3	5	92	0.002	–	–	0.002	0.026	0.003
	159.4	0	2	98	0.000	–	–	0.000	0.012	0.000
	180.3	0	0	100	0.000	–	–	0.000	0.000	0.000
A_2	5.4	81	9	10	0.589	–	0.295	–	–	0.386
	23.1	12	71	17	0.012	–	0.006	–	–	0.008
	31.3	86	5	9	0.008	–	0.004	–	–	0.005
	37.4	17	57	26	0.086	–	0.043	–	–	0.056
	61.2	21	11	68	0.108	–	0.054	–	–	0.071
	70.9	4	16	80	0.002	–	0.001	–	–	0.001
	71.8	49	35	16	0.000	–	0.000	–	–	0.000
	79.4	14	1	85	0.001	–	0.000	–	–	0.000
	88.4	10	52	38	0.017	–	0.008	–	–	0.011
	105.6	4	29	67	0.012	–	0.006	–	–	0.008
	132.5	2	5	93	0.001	–	0.001	–	–	0.001
	160.9	0	4	96	0.000	–	0.000	–	–	0.000
	180.0	0	0	100	0.000	–	0.000	–	–	0.000
B_2	19.0	11	30	59	0.026	–	0.013	–	0.000	0.017
	34.9	6	78	16	0.067	–	0.034	–	0.018	0.044
	44.6	1	88	11	0.010	–	0.005	–	0.039	0.007
	51.5	2	26	72	0.011	–	0.005	–	0.006	0.007
	61.3	70	14	16	0.001	–	0.000	–	0.003	0.000
	65.6	1	20	79	0.036	–	0.018	–	0.003	0.024
	82.2	71	9	20	0.024	–	0.012	–	0.019	0.016
	87.8	13	25	62	0.010	–	0.005	–	0.008	0.007
	106.5	21	2	77	0.063	–	0.032	–	0.089	0.042
	128.4	3	7	90	0.002	–	0.001	–	0.009	0.001
	156.9	0	0	100	0.051	–	0.026	–	0.013	0.033
	180.6	0	0	100	0.002	–	0.001	–	0.000	0.001

Table S2: Calculated wavenumbers of the lowest energy modes of phenytoin form II. Symbols indicate symmetry and correspond to those used in Figure 6 of main text. For each mode we also report translational, rotational and internal components (%T, %R and %I) and infrared and Raman (Figure 6) intensities for a powder. Intensities of inactive modes for the given symmetry are indicated by hyphens. The eigenvector of the mode with imaginary frequency (indicated in bold) is represented in Figure 5.

Pc							$P2_1/c$						
Sym	Freq (cm^{-1})	%T	%R	%I	Powder I_{IR}	Powder I_{Raman}	Sym	Freq (cm^{-1})	%T	%R	%I	Powder I_{IR}	Powder I_{Raman}
A'	20.9	82	8	10	0.000	1.000	A_g	20.3	88	0	12	–	0.731
	31.6	16	60	24	0.006	0.456		28.4	8	76	16	–	0.267
	34.5	6	68	26	0.011	0.078		38.2	16	76	8	–	0.038
	38.1	6	84	10	0.029	0.048		50.5	72	8	20	–	0.055
	50.2	66	10	24	0.015	0.124		64.8	0	52	48	–	0.081
	56.8	4	46	50	0.046	0.061		71.5	0	12	88	–	0.287
	58.2	10	64	26	0.651	0.191		80.7	40	0	60	–	0.151
	65.6	2	62	36	0.077	0.048		84.5	12	12	76	–	0.637
	69.7	0	40	60	0.124	0.282		95.7	56	12	32	–	0.039
	71.8	8	30	62	0.030	0.077		109.6	0	40	60	–	0.215
	77.6	34	4	62	0.021	0.106		131.5	0	4	96	–	0.082
	82.6	44	8	48	0.045	0.041		143.0	0	0	100	–	0.148
	84.2	12	6	82	0.013	0.065		187.3	0	0	100	–	0.016
	88.5	4	8	88	0.013	0.070	B_u	**32.6** i	0	16	84	0.090	–
	94.8	20	18	62	0.011	0.481		41.6	0	88	12	0.041	–
	96.2	48	10	42	0.005	0.080		61.1	0	56	44	0.477	–
	104.0	24	20	56	0.123	0.036		69.3	8	68	24	0.077	–
	111.9	2	36	62	0.024	0.238		77.9	16	20	64	0.016	–
	126.7	0	6	94	0.439	0.050		82.9	44	0	56	0.017	–
	133.5	0	6	94	0.098	0.060		88.4	0	12	88	0.021	–
	141.7	0	0	100	0.526	0.157		103.1	28	24	48	0.118	–
	146.2	0	2	98	1.000	0.048		127.1	0	4	96	0.366	–
	187.9	0	0	100	0.002	0.022		144.4	0	0	100	1.000	–
	188.2	0	0	100	0.633	0.000		187.5	0	0	100	0.437	–
A''	31.7	82	2	16	0.000	0.160	B_g	38.0	84	8	8	–	0.067
	34.0	24	68	8	0.002	0.018		42.9	80	12	8	–	0.200
	35.2	60	22	18	0.013	0.024		50.2	44	48	8	–	0.135
	39.9	64	18	18	0.008	0.009		70.2	8	4	88	–	1.000
	42.2	60	30	10	0.001	0.159		73.8	4	88	8	–	0.145
	45.4	34	60	6	0.000	0.009		76.3	32	52	16	–	0.006
	53.9	28	42	30	0.003	0.142		89.5	8	4	88	–	0.348
	67.6	2	30	68	0.037	0.049		104.9	0	8	92	–	0.724
	68.9	6	78	16	0.011	0.126		107.5	8	16	76	–	0.170
	77.1	36	48	16	0.005	0.018		112.9	8	12	80	–	0.466
	83.8	10	14	76	0.018	0.245		140.7	0	0	100	–	0.018
	88.2	6	2	92	0.000	0.265		183.2	16	36	48	–	0.022
	95.1	8	14	78	0.005	0.125		189.6	0	4	96	–	0.003
	100.8	4	12	84	0.001	0.113	A_u	35.0	36	44	20	0.005	–
	105.1	6	10	84	0.032	0.046		37.1	52	24	24	0.012	–
	105.5	4	8	88	0.002	0.582		38.5	60	32	8	0.003	–
	111.9	10	6	84	0.008	0.296		46.2	0	68	32	0.001	–
	117.8	6	6	88	0.068	0.599		67.9	4	36	60	0.027	–
	122.7	0	20	80	0.008	0.277		88.7	12	12	76	0.016	–
	140.9	0	0	100	0.000	0.017		100.5	4	12	84	0.004	–
	145.3	0	2	98	0.008	0.010		109.2	8	0	92	0.092	–
	181.8	18	36	46	0.211	0.019		111.4	4	20	76	0.005	–
	186.0	20	36	44	0.260	0.008		143.6	0	0	100	0.002	–
	189.5	0	0	100	0.000	0.002		184.2	16	36	48	0.350	–
	190.4	2	6	92	0.029	0.002		189.1	0	4	96	0.027	–
